# Eligible but Untreated: Gaps in Statin Therapy for Type 1 Diabetes Under the National Institute for Health and Care Excellence (NICE) Guidelines

**DOI:** 10.7759/cureus.98557

**Published:** 2025-12-06

**Authors:** Muhammad Haroon Riasat, Samraiz Nafees, Sakshi Malik, Salwa Rahman, Keimee Lopez, Omaya Abdulhadi, Neve Wright, Rohan Chikhal, Sufyan Benamer

**Affiliations:** 1 Diabetes and Endocrinology, Hull University Teaching Hospitals NHS Trust, Hull, GBR; 2 Internal Medicine, York and Scarborough NHS Trust, Scarborough, GBR; 3 Internal Medicine, Hull University Teaching Hospitals NHS Trust, Hull, GBR; 4 Vascular Surgery, Hull University Teaching Hospitals NHS Trust, Hull, GBR

**Keywords:** cardiovascular disease, nephropathy, nice guidelines, primary prevention, retinopathy, statin therapy, type 1 diabetes mellitus

## Abstract

Background and objective

Cardiovascular disease (CVD) represents a principal contributor to premature morbidity and mortality in individuals with type 1 diabetes mellitus (T1DM). Effective lipid management, including the use of statin therapy, offers substantial potential to mitigate long‑term vascular risk, particularly among patients with established microvascular complications or long disease duration. This study aimed to assess the extent to which statin prescribing practices in a large healthcare trust align with the current National Institute for Health and Care Excellence (NICE) guidelines.

Methods

We performed a retrospective review of electronic health records for T1DM patients under the care of a single healthcare trust. Patients were stratified into two groups based on age: ≥40 years and <40 years. According to NICE criteria, statin therapy is indicated for patients aged ≥40 years, or those of any age with diabetes duration >10 years, diabetic nephropathy, or other modifiable cardiovascular risk factors. The main outcome measure was the proportion of eligible patients who had been prescribed statin therapy. Data were analyzed using descriptive statistics.

Results

Among 1,229 patients aged ≥40 years, 896 (73%) were receiving statins, leaving 333 (27%) without therapy despite being eligible. In the <40 years age group, 192 patients met NICE indications for statin use. Prescription rates were notably lower in this cohort: six (35%) for those with nephropathy, 2two(15%) among those with retinopathy, and 75 (47%) for patients with both risk factors. Overall, only 83 (43%) of eligible patients under 40 were prescribed statins.

Conclusions

Our findings reveal a significant gap between guideline recommendations and real-world prescribing patterns, particularly among younger T1DM patients with additional CVD risk factors. Given the elevated cardiovascular risk in this population, strategies to enhance clinician awareness and improve adherence to NICE guidance are urgently needed to optimize primary prevention.

## Introduction

Diabetes mellitus, a chronic metabolic disorder, has seen a significant rise in prevalence globally. In the United Kingdom, 4.7 million people were affected as of 2019, with type 1 diabetes mellitus (T1DM) accounting for 8% of cases and the highest prevalence observed in individuals aged 35-60 years. Cardiovascular disease (CVD) remains the leading cause of morbidity and mortality among patients with diabetes, with evidence indicating a two-fold increase in mortality rates attributable to CVD in this population [1-4]. Despite advances in glycemic control, which have proven effective in reducing microvascular complications, macrovascular outcomes such as CVD remain inadequately addressed by glycemic management alone [5].​

Dyslipidemia is a well-established modifiable risk factor for CVD, and its management is crucial in the primary prevention of cardiovascular events in patients with T1DM. The presence of diabetes-related microvascular complications, such as nephropathy and retinopathy, further amplifies the risk of CVD by two to three times [6-9]. In response, the National Institute for Health and Care Excellence (NICE) recommends statin therapy for primary prevention in adults with T1DM who possess additional risk factors, including age over 40 years, diabetes duration exceeding 10 years, established nephropathy, or other modifiable risk factors such as obesity or smoking [10,11].​​

Despite the availability of clear guidance, real-world adherence to these recommendations remains variable. Marked variation in prescribing practices across regions and between NHS trusts has been reported, reflecting differences in clinician awareness, local pathways, and implementation of NICE guidance, highlighting the importance of evaluating adherence within our trust [12]. This study, therefore, aims to retrospectively assess adherence to NICE guidelines for primary cardiovascular prevention in patients with T1DM within a large NHS trust, to identify practice gaps and potential areas for quality improvement.

## Materials and methods

A retrospective clinical audit was conducted in the Diabetes and Endocrinology Department at Hull Royal Infirmary to evaluate adherence to recommended statin prescribing practices in adults with T1DM who met the NICE criteria for primary CVD prevention [13]. This project was part of a quality improvement initiative within the trust to evaluate preventive cardiovascular care in this population.

The departmental diabetes database served as the primary data source for patient identification. Unique NHS numbers were used to extract anonymized patient data, which were then cross‑referenced against electronic clinical records to confirm T1DM diagnosis and assess eligibility for statin therapy. Eligibility was determined according to established clinical risk factors, including age ≥ 40 years, diabetes duration exceeding 10 years, established microvascular disease (nephropathy or retinopathy), or the presence of additional modifiable risk factors such as hypertension, smoking, or dyslipidemia (Table 1). To ensure data accuracy, each record was reviewed for consistency between electronic systems and supporting clinical documentation.

**Table 1:** Summary of key variables and definitions used in the audit ACR: albumin-to-creatinine ratio; KDIGO: the Kidney Disease Improving Global Outcomes; eGFR: estimated glomerular filtration rate

Variable	Definition/criteria
Age	≥40 years (Cohort A); <40 years (Cohort B)
Diabetes duration	>10 years
Nephropathy	ACR ≥3 mg/mmol on ≥2 occasions (per KDIGO guidelines) or eGFR <60 mL/min/1.73m²
Retinopathy	Any diabetic retinopathy (R1 or above) per the UK National Screening Programme classification
Other risk factors	Hypertension (BP ≥140/90 mmHg or treated), current smoking, dyslipidemia (LDL ≥3 mmol/L or treated)

**Figure 1:**
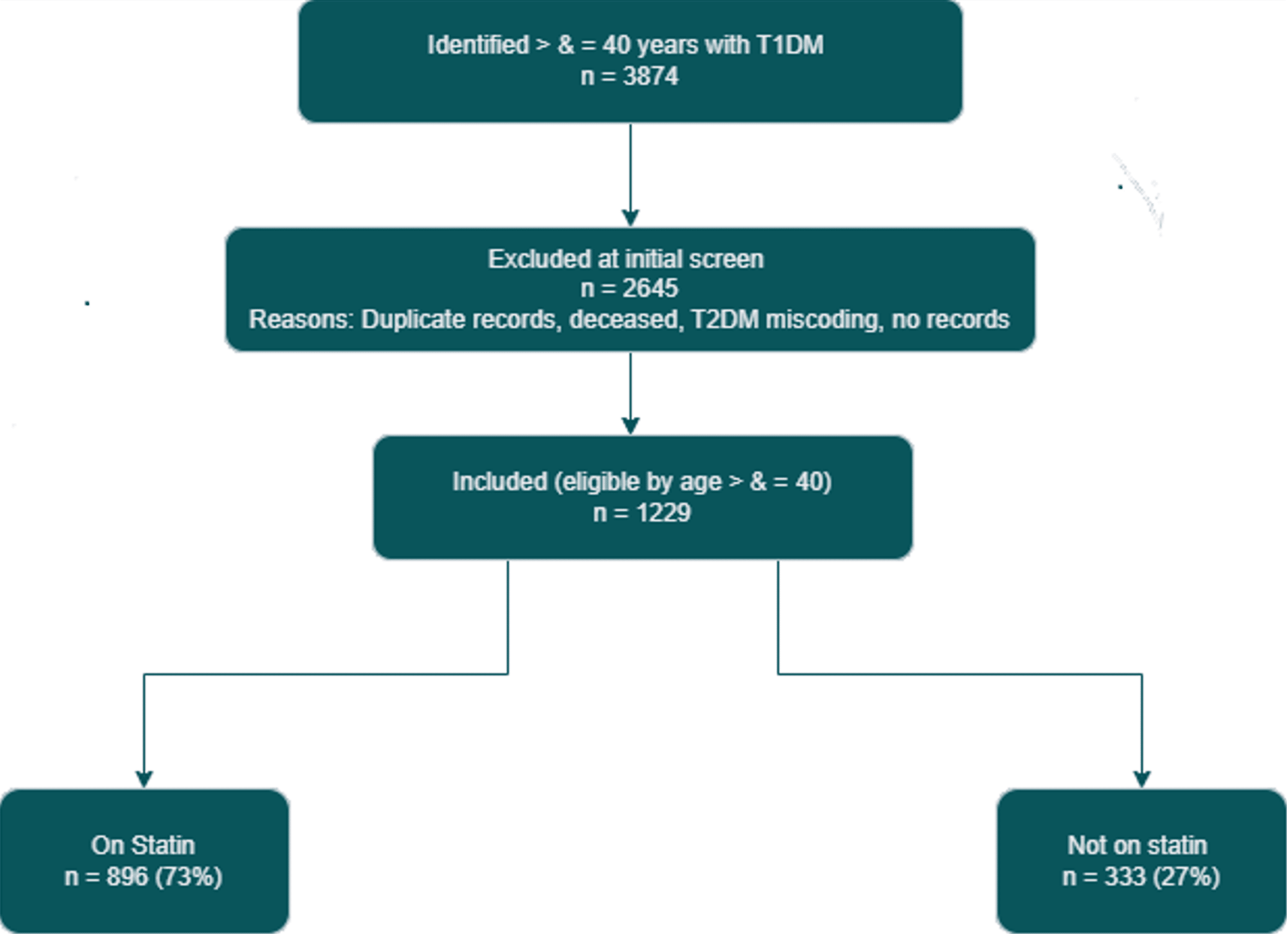
Cohort A: patients aged ≥40 years Diagram assessing adherence to NICE criteria for statin prescribing in adults with type 1 diabetes for primary prevention of cardiovascular disease [13] Flow diagram produced using draw.io (diagrams.net, a free web application) [14] NICE: National Institute for Health and Care Excellence; T1DM: type 1 diabetes mellitus; T2DM: type 2 diabetes mellitus

**Figure 2:**
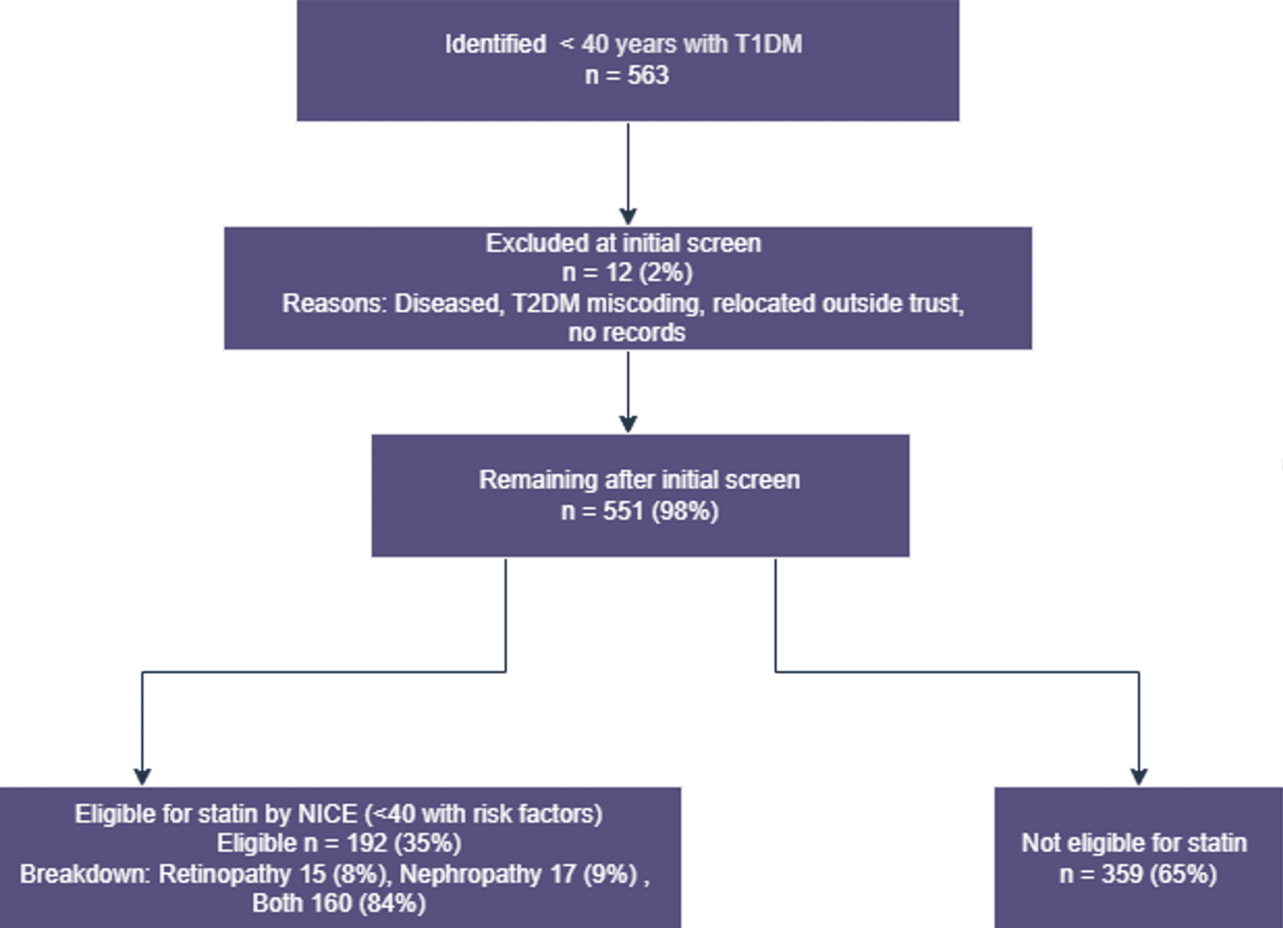
Cohort B: patients aged <40 years Diagram assessing adherence to NICE criteria for statin prescribing in adults with type 1 diabetes for primary prevention of cardiovascular disease [13] Flow diagram produced using draw.io (diagrams.net, a free web application) [14] NICE: National Institute for Health and Care Excellence; T1DM: type 1 diabetes mellitus; T2DM: type 2 diabetes mellitus

Patients were excluded if they met any of the following criteria: documented duplication of records, death before audit review, confirmed diagnosis of type 2 diabetes mellitus (T2DM), or incomplete/missing data in the relevant clinical fields. Following the application of these criteria, eligible cases were stratified into two analytical cohorts. Cohort A comprised individuals aged ≥ 40 years (Figure 1) [14], and Cohort B included those aged < 40 years (Figure 2) [14]. Within each cohort, risk‑defining complications were further subclassified according to the presence of nephropathy, retinopathy, or a combination of both. For each subgroup, the current statin prescription status, treatment discontinuation, and documented intolerance were recorded.

Data extraction was conducted using the Lorenzo hospital electronic medical record system, supplemented by GP Connect to verify community prescriptions and updated medication summaries. Additional data sources included scanned clinic letters, consultant correspondence, and laboratory results confirming the presence of albuminuria or evidence of diabetic retinopathy on retinal screening databases. To ensure reliability and minimize selection bias, each record underwent dual verification by two independent audit members, with a third reviewer adjudicating discrepancies during consensus meetings. The audit team consisted of registrars, senior house officers, and foundation doctors under consultant supervision, ensuring rigorous data governance and compliance with trust audit procedures.

Data management and analysis were performed using Microsoft Excel 2021. Categorical data were summarized using absolute frequencies and proportions, while continuous variables (such as age and diabetes duration) were expressed as means with corresponding ranges. Descriptive statistical methods were employed to explore variation in prescribing rates between cohorts and complication groups. Results were displayed in flow diagrams, cross‑tabulated summaries, and comparative visual charts to highlight prescribing trends, missed opportunities for therapy, and potential gaps in adherence to the NICE primary prevention framework.

Ethical approval was not required as this audit constituted a trust‑approved service evaluation. Patient confidentiality was maintained throughout in accordance with local governance regulations, and no identifiable information was exported outside secure hospital systems.

## Results

**Figure 3:**
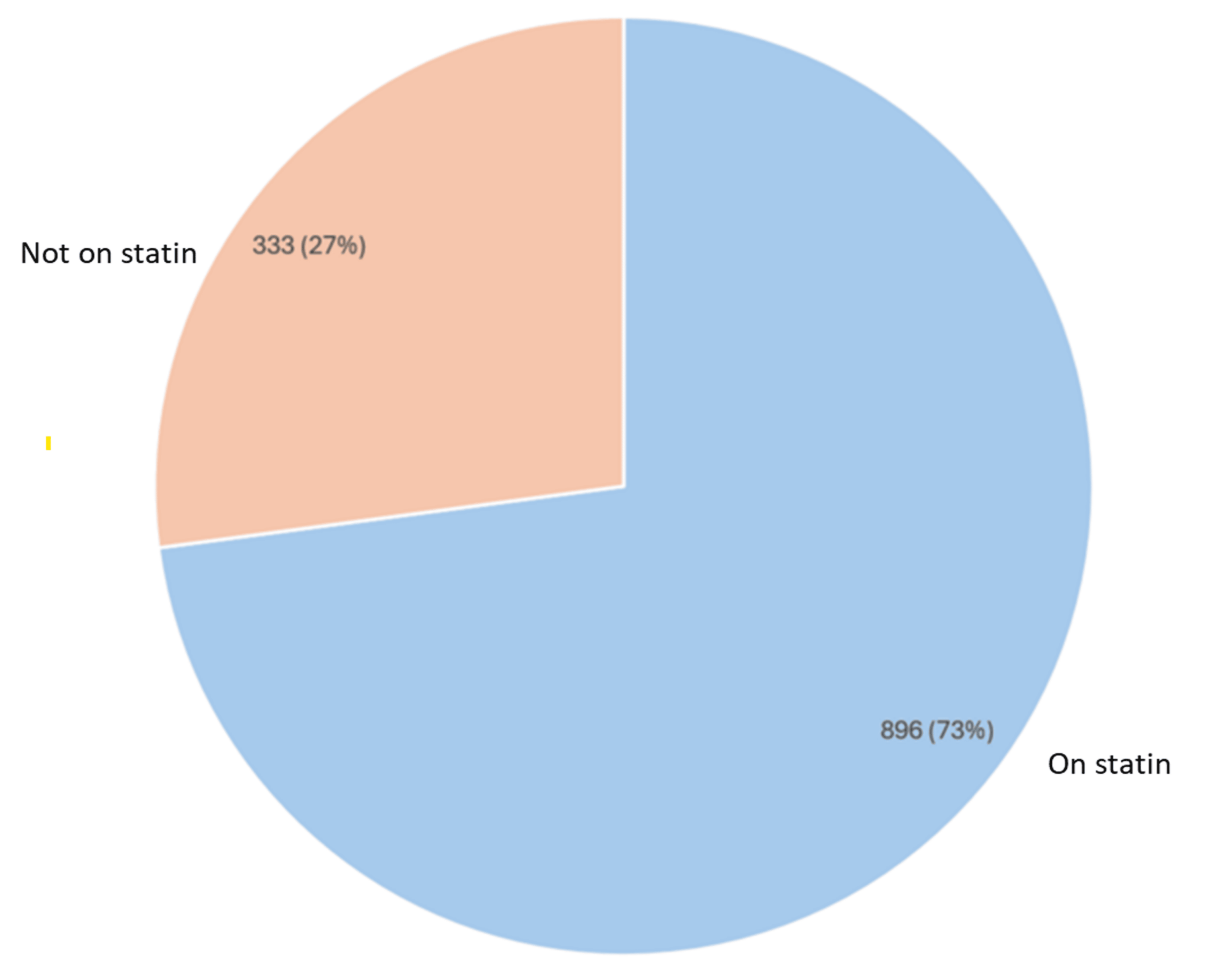
Statin prescription status in patients aged 40 years and older with type 1 diabetes mellitus Chart assessing adherence to NICE criteria for statin prescribing in adults with type 1 diabetes for primary prevention of cardiovascular disease [13] Pie chart generated using Microsoft Excel 2021 [15] NICE: National Institute for Health and Care Excellence

A total of 3,874 patients aged 40 years and older were initially identified. Following the exclusion of duplicates, deceased individuals, those with a T2DM diagnosis, and those with missing records, 1,229 patients remained for the final analysis. Among these, 896 (73%) were prescribed statins in line with NICE recommendations, whereas 333 (27%) were not receiving statin therapy despite meeting the eligibility criteria (Figure 3) [14]. This indicates a significant gap in guideline adherence among the older cohort.

**Figure 4:**
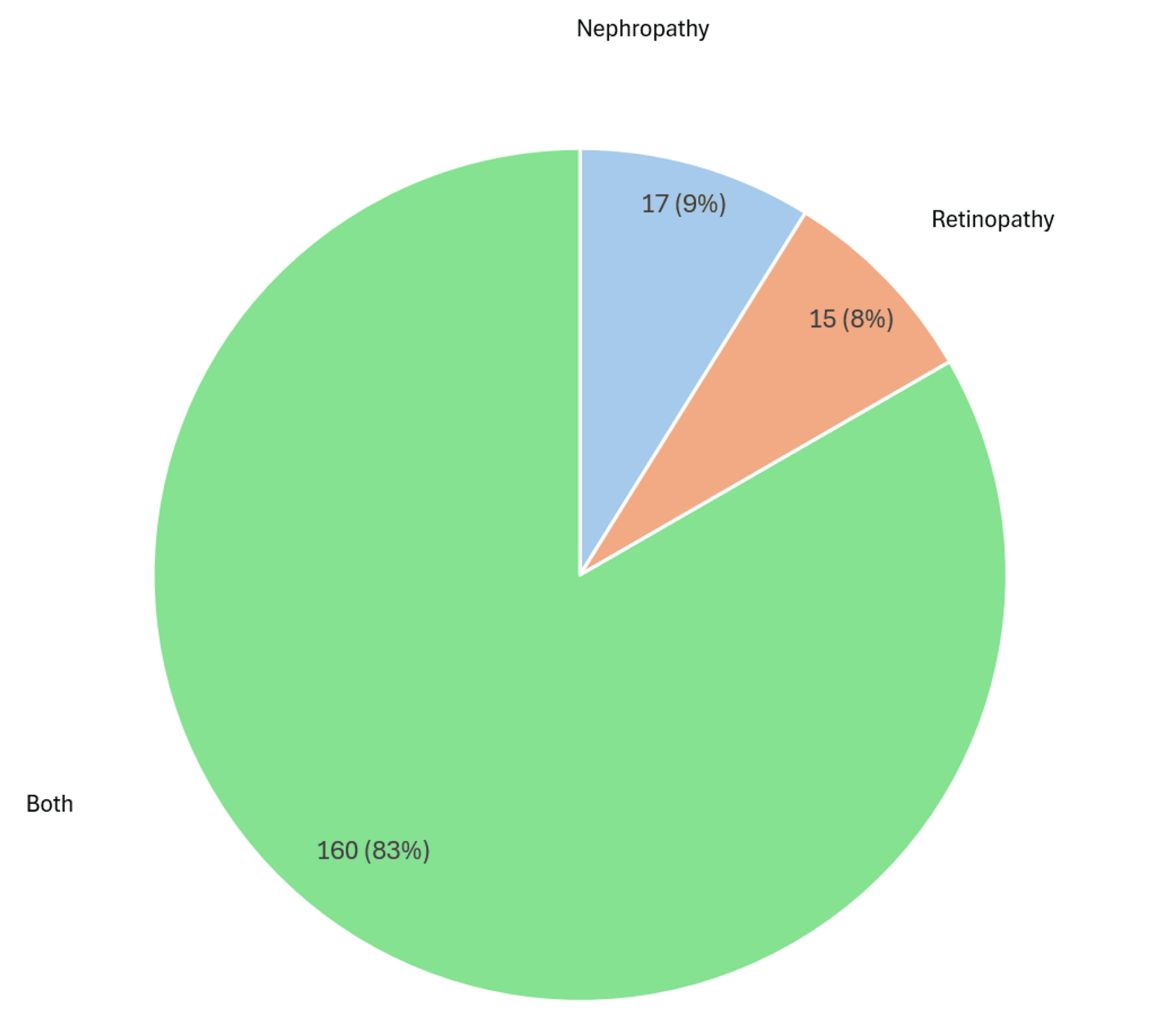
Indications for statin therapy in type 1 diabetes patients by retinopathy and nephropathy status Chart assessing adherence to NICE criteria for statin prescribing in adults with type 1 diabetes for primary prevention of cardiovascular disease [13] Pie chart generated using Microsoft Excel 2021 [15] NICE: National Institute for Health and Care Excellence

In the cohort of patients younger than 40 years, 563 individuals were screened, with exclusions applied for deceased status, a diagnosis of T2DM, relocation outside the trust, and missing data. The final analysis included 192 (34%) patients who had a documented indication for statin therapy based on the presence of retinopathy (n = 15, 8%), nephropathy (n = 17 , 9%), or both risk factors (n = 160 , 83%) (Figure 4) [14].

**Figure 5:**
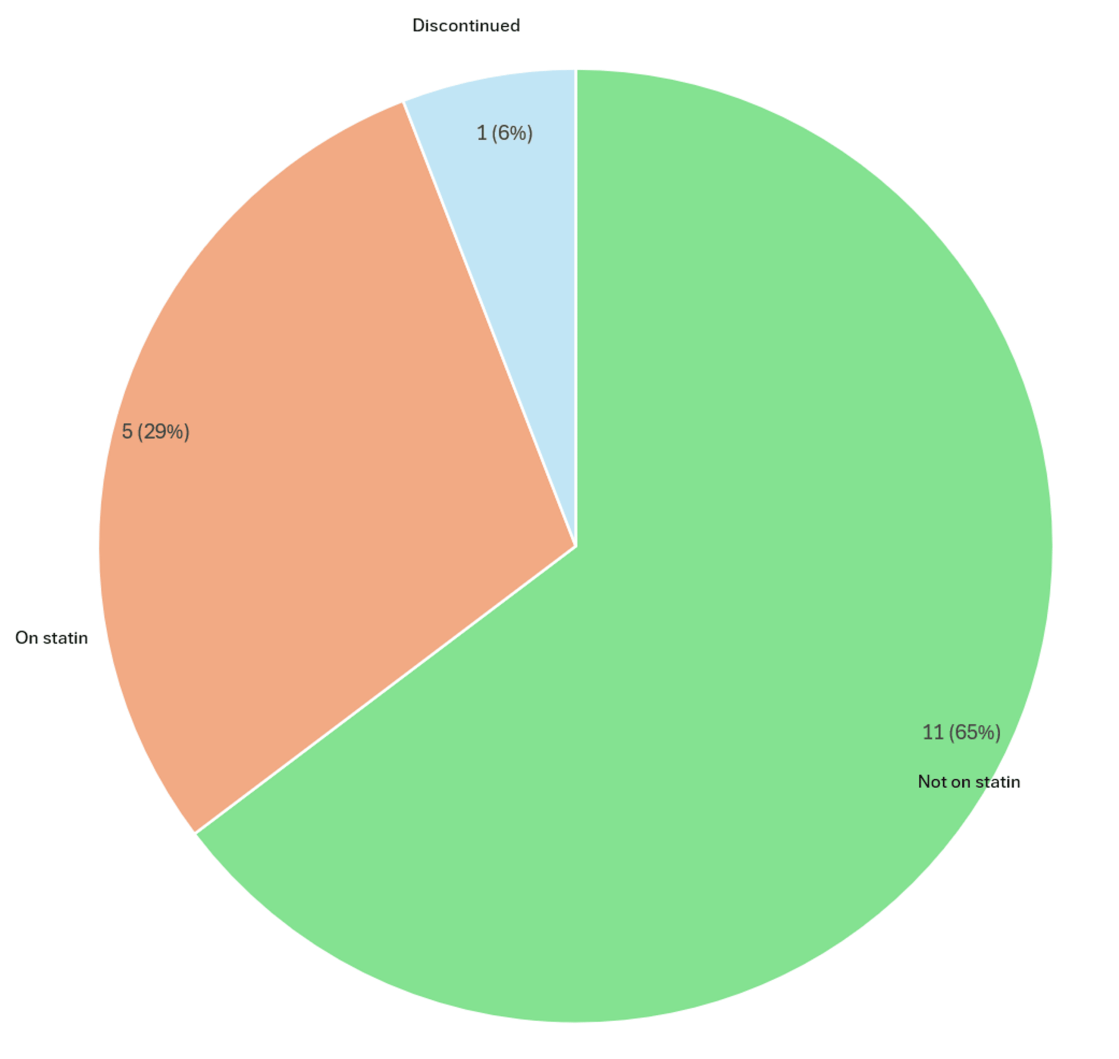
Statin therapy status among patients with nephropathy in type 1 diabetes Chart assessing adherence to NICE criteria for statin prescribing in adults with type 1 diabetes for primary prevention of cardiovascular disease [13] Pie chart generated using Microsoft Excel 2021 [15] NICE: National Institute for Health and Care Excellence

**Figure 6:**
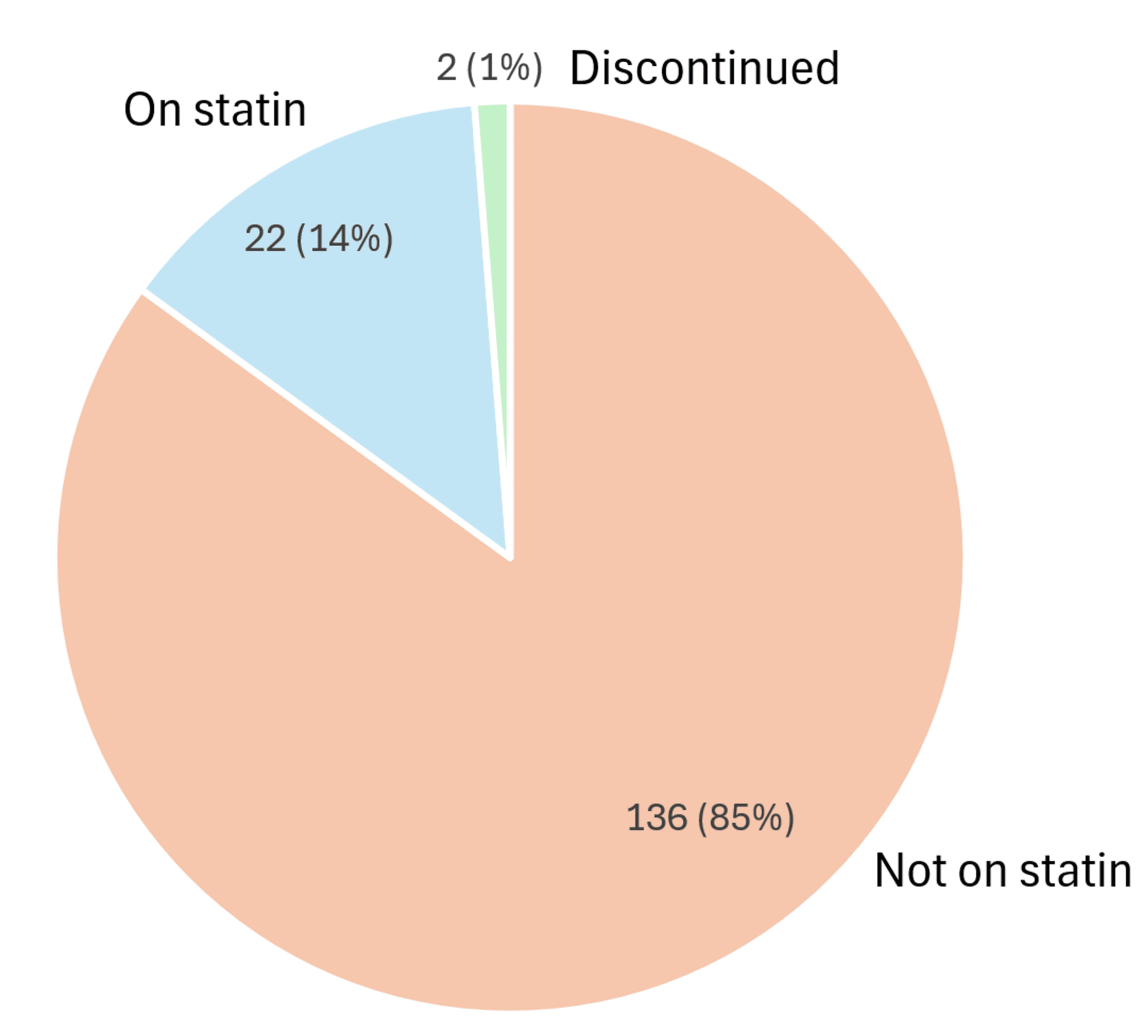
Statin therapy status among patients with retinopathy in type 1 diabetes Chart assessing adherence to NICE criteria for statin prescribing in adults with type 1 diabetes for primary prevention of cardiovascular disease [13] Pie chart generated using Microsoft Excel 2021 [15] NICE: National Institute for Health and Care Excellence

**Figure 7:**
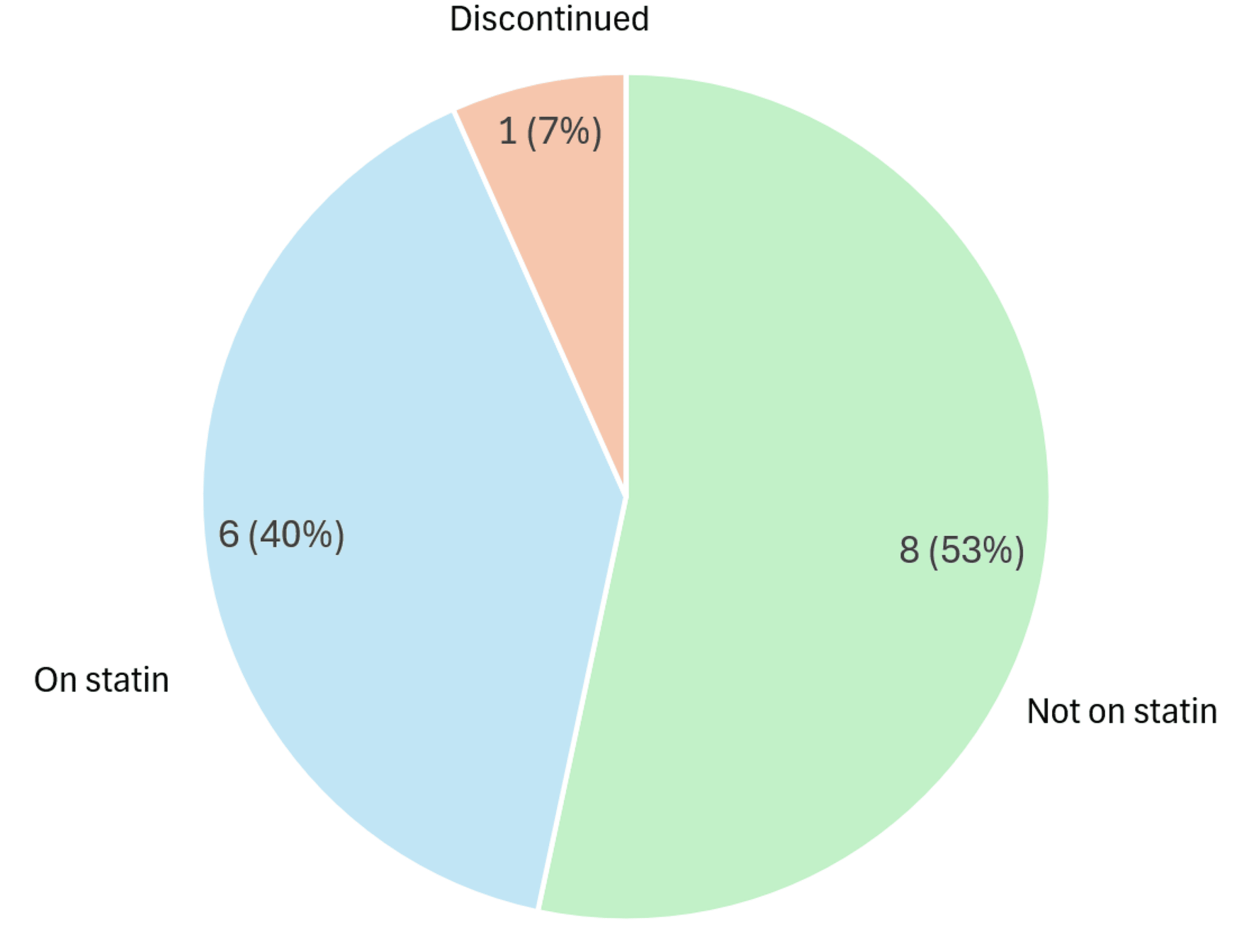
Statin therapy status among patients with both nephropathy and retinopathy in type 1 diabetes Chart assessing adherence to NICE criteria for statin prescribing in adults with type 1 diabetes for primary prevention of cardiovascular disease [13] Pie chart generated using Microsoft Excel 2021 [15] NICE: National Institute for Health and Care Excellence

Within the nephropathy subgroup, 11 (65%) were not on statin therapy, five (29%) were prescribed statins, and one (6%) had discontinued statins. Among those with retinopathy, 136 (85%) were not receiving statins, 22 (14%) were prescribed statins, and two patients (1%) had discontinued therapy. For patients with both nephropathy and retinopathy, eight (53%) were not on statins, six (40%) were prescribed statins, and one (7%) had discontinued therapy (Figures 5-7) [14].

These findings reveal a consistent trend of underutilization of statin therapy among high-risk T1DM patients, particularly in younger individuals and those with microvascular complications. The data highlight that a substantial proportion of eligible patients remain untreated, despite clear evidence-based guidelines and the well-documented benefits of statin therapy in reducing cardiovascular risk.

## Discussion

The results of this study highlight a significant gap between evidence-based recommendations and real-world clinical practice in the primary prevention of CVD among patients with T1DM. While statin therapy is widely recognized as an effective intervention for reducing cardiovascular risk, particularly in high-risk populations, adherence to NICE guidelines remains suboptimal. In the cohort aged 40 years and older, nearly one in four eligible patients were not receiving statins, exposing them to preventable cardiovascular events [16,17]. This is particularly concerning given the established association between diabetes and elevated CVD-related mortality, as well as the persistent burden of CVD in the UK, which accounts for a quarter of all deaths annually [18].

The situation is even more pronounced among younger patients with T1DM who present with microvascular complications [19,20]. Despite the presence of nephropathy or retinopathy - both strong predictors of future cardiovascular events - statin therapy was under-prescribed, with up to 85% of patients with retinopathy and 65% with nephropathy not receiving appropriate lipid-lowering treatment. These findings are consistent with previous studies demonstrating that microvascular disease substantially increases the risk of CVD and that early intervention is critical for favorable long-term outcomes [21-23]

Several factors may contribute to these gaps in care, including the lack of electronic medical record alerts for statin eligibility, delays in outpatient follow-up for risk assessment, and limited clinician familiarity with specific NICE lipid guidelines for T1DM. Patient preferences, concerns about statin intolerance, and broader system-level prescribing barriers may also play a role. The audit also highlights the importance of regular risk stratification and proactive management, particularly in younger patients and those with evolving microvascular disease. Dissemination of audit findings, targeted educational interventions for clinicians, and system-wide reminders may help bridge the gap between guidelines and practice. Future research should examine the underlying reasons for non-adherence and evaluate the impact of targeted quality-improvement initiatives on statin prescribing rates and cardiovascular outcomes.

A key limitation of this service evaluation is reliance on routinely collected records, which may lead to diagnostic misclassification and omit prescriptions initiated outside the trust (information bias). We lacked granular reasons for non-prescribing, and the exclusion categories did not have verified counts per reason. As this was a single-trust, retrospective audit, the results may not be generalizable to other settings or account for changes over time.

## Conclusions

This audit highlights a substantial treatment gap in the prescription of statin therapy among adults with T1DM who meet NICE eligibility criteria, particularly in younger patients and those with microvascular complications. Despite clear evidence of cardiovascular risk in this population, a significant proportion remain untreated, underscoring missed opportunities for primary prevention. These findings suggest the need for stronger implementation strategies, including systematic identification of eligible patients, active collaboration with regional general practitioners, and use of electronic prescribing prompts at the point of care. Reinforcing awareness through targeted educational sessions and clinical reminder posters, alongside routine re‑audits, will help sustain improvements and promote consistent adherence to guideline‑based cardiovascular risk reduction in this high‑risk group.
